# Special Issue “Organophosphorus Chemistry: A New Perspective”

**DOI:** 10.3390/molecules28124752

**Published:** 2023-06-14

**Authors:** Jakub Adamek

**Affiliations:** 1 Department of Organic Chemistry, Bioorganic Chemistry and Biotechnology, Silesian University of Technology, B. Krzywoustego 4, 44-100 Gliwice, Poland; jakub.adamek@polsl.pl; Tel.: +48-032-237-1724; Fax: +48-032-237-2094; 2 Biotechnology Center, Silesian University of Technology, B. Krzywoustego 8, 44-100 Gliwice, Poland

The European Chemical Society (EuChemS) and the European Parliament (Science and Policy Workshop, 25 May 2023) recognize phosphorus as one of the key chemical elements in daily life. It is not only a component of the human body but also a foundation of the agrochemical industry. Today, it is often said that we are living in “the golden age of phosphorus chemistry”. In this context, organophosphorus chemistry is also gaining importance as one of the fastest-growing branches of organic chemistry. In the laboratory, phosphorus-containing compounds (also called P-compounds) are widely used as reagents (starting materials, precursors of active intermediates such as ylides or iminium-type cations, etc.), catalysts (PTC, organocatalysis), and solvents (PILs) [1,2,3,4]. Due to the interesting properties of P-compounds (especially their biological activity), they are used on a large scale in medicine (e.g., bone disorder drugs, anticancer and antiviral agents, and antihelminthics in veterinary applications), agriculture (e.g., pesticides), and industry (e.g., production of lubricants or plastic materials) [5,6,7]. However, in the age of much-needed care for the natural environment, we face new challenges. Innovative approaches to the synthesis and isolation of P-compounds (taking into account the aspects of green chemistry and sustainability), followed by their responsible use and disposal (neutralization), may prove crucial in the near future.

In this Special Issue, seven original research articles and three reviews covering aspects of recent advances in the synthesis, transformation, and properties of organophosphorus compounds were published.

The first two articles concern phosphonium salts and the properties of the phosphonium moiety [8,9]. In a review article, Adamek et al. collected information on the synthesis and reactivity of 1-aminoalkylphosphonium derivatives [8]. As shown, these types of compounds can be considered not only as smart synthetic equivalents of *N*-acyliminium-type cations in the α-amidoalkylation reaction but also as convenient reagents in cyclizations or effective precursors of ylides in the Wittig reaction. In turn, Grymel et al. described the synthesis and, subsequently, the cytotoxicity and antibacterial activity of triphenylphosphonium derivatives of betulin [9]. In total, nine new molecular hybrids of betulin with covalent linkage of the alkyltriphenylphosphonium moiety to the parent skeleton were obtained, with good to excellent yields. They showed high cytotoxicity (greater than natural betulin) toward the cell lines tested (HCT 116 and MCF-7), as well as antimicrobial properties against the Gram-positive reference *Staphylococcus aureus ATCC 25923* and *Staphylococcus epidermidis ATCC 12228* bacteria.

The next two articles address bisphosphoric systems, together with their synthesis and application [10,11]. Kuźnik et al. disclosed a simple and effective strategy for the synthesis of *N*-protected bisphosphoric analogs of protein and non-protein α-amino acids [10]. Indeed, the method based on the three-component reaction of 1-(*N*-acylamino)-1-ethoxyphosphonates with triphenylphosphonium tetrafluoroborate and triethyl phosphite allowed for the acquisition of 14 compounds with yields in the range of 40–96%. The proposed methodology can also be used in the synthesis of unsymmetric bisphosphoric compounds via the sequential formation of C-P bonds with different phosphorus nucleophiles. The importance of research on bisphosphonates was also emphasized by Demadis et al. in their manuscript on drug-inclusive, inorganic–organic hybrid systems for the controlled release of zoledronate [11]. Two coordination polymers containing alkaline earth metal ions (Sr^2+^ and Ba^2+^) and zoledronate (ZOL, the anti-osteoporotic drug) were synthesized and characterized. On the basis of the conducted studies, the influences of the type of cation on both the initial rate of drug release and the final value of the plateau release were determined.

The other articles are related to phosphonate compounds, their synthesis, reactivity, and biological properties [12,13,14,15,16]. Moilanen et al. prepared an interesting review article about the applications of α-aminophosphonates, -phosphinates, and -phosphine oxides as extraction and precipitation agents for rare earth metals, thorium, and uranium [12]. The authors described the most important methods for the synthesis of the abovementioned organophosphorus compounds and characterized their ability as extractants and precipitation agents. Some future perspectives related to the tunability of the solubility and coordination affinity of the α-amino-functionalized organophosphorus compounds were also discussed. Olszewski et al. described the deamination of 1-aminoalkylphosphonic acids in reaction with HNO_2_ [13]. Mechanistic research and analysis of the obtained products allowed the authors to propose a plausible mechanism reaction with the formation of 1-phosphonoalkylium ions as reactive intermediates. Vicario et al. presented a general strategy for the synthesis of a wide family of α-aminophosphonate analogs of aspartic acid with tetrasubstituted carbons via the aza-Reformatsky reaction of α-iminophosphonates, generated from α-aminophosphonates [14]. In total, more than 20 such compounds were synthesized. Their cytotoxicity was also evaluated, and the structure–activity profile was determined. A one-pot lithiation–phosphonylation protocol to prepare heteroaromatic phosphonic acids was reported by Chmielewska et al. [15]. The scope of application and limitations of the proposed method were explored. The antiproliferative activity of the compounds obtained was also tested. Kiełbasiński and Janicki described the application of alkyl di-(1,1,1,3,3,3-hexafluoroisopropyl)phosphonoacetates in the highly Z-selective Horner–Wadsworth–Emmons olefination as modified Still–Gennari-type reagents [16]. Excellent results, with an up to a 98:2 Z:E product ratio and up to quantitative yield, were achieved using the abovementioned reagents in the olefination of aromatic aldehydes.

Finally, the review article prepared by Bałczewski et al. introduces readers to the chemistry of linearly fused aromatics, called acenes [17]. This study is not only a retrospective investigation but also a presentation of the current state of knowledge on the synthesis, properties, and applications of phosphorus (P^III^, P^IV^, P^V^)-substituted acenes.

In conclusion, organophosphorus chemistry continues to attract the unwavering interest of many research groups. The level of the research presented is high, and its subject matter attracts great attention, as evidenced by increasing metrics (citations, views). Therefore, I would like to thank all the authors who chose to report their results in this Special Issue and acknowledge the contributions of the Academic Editors: Gabriele Micheletti, Constantina Papatriantafyllopoulou, Erika Bálint, and György Keglevich; all the peer reviewers; and the members of the Editorial Team, especially Marlene Zhang. Your support has been invaluable.
